# Effects of Tea Polyphenols on Post-Weaning Meat Quality and Antioxidant Status in Lambs

**DOI:** 10.3390/ani15162414

**Published:** 2025-08-18

**Authors:** Yuxin Bai, Jialin Wang, Jian Ma, Chunmei Du, Fuquan Yin

**Affiliations:** College of Coastal Agriculture Science, Guangdong Ocean University, Zhanjiang 524088, China; baiyuxinbb@163.com (Y.B.); 18180871933@163.com (J.W.); crazyma0411@163.com (J.M.)

**Keywords:** tea polyphenols, weaned lambs, meat quality, muscle fatty acids, antioxidant activity

## Abstract

In this present study, we aimed to investigate the effects of adding tea polyphenols to feed on the meat quality, muscle antioxidant capacity, and muscle fatty acid composition of lambs. Forty healthy 45-day-old weaned lambs with similar body weights (average initial weight 9.1 ± 0.8 kg) were randomly divided into four treatment groups (*n* = 10) for a 45-day trial. The control group (CON) was fed a basal diet, while the other three groups (TP2, TP4, and TP6) received the basal diet supplemented with 2, 4, and 6 g/kg of tea polyphenols, respectively. Supplementation with tea polyphenols significantly reduced the cooked meat rate of biceps femoris in the TP2 and TP6 groups, and significantly increased the pH values of triceps brachii at 0 h and 24 h in the TP6 group (*p* < 0.05). Increased activities of total superoxide dismutase in the longissimus dorsi and glutathione peroxidase in biceps femoris, significantly decreased contents of hydrogen peroxide in the biceps femoris and triceps brachii, and significantly increased activity of catalase in triceps brachii were observed (*p* < 0.05). TP4 and TP6 significantly increased the activities of the total antioxidant capacity in biceps femoris and glutathione peroxidase in triceps brachii, and significantly decreased the concentration of malondialdehyde in biceps femoris (*p* < 0.05). The dietary tea polyphenols significantly increased the contents of C16-1, C18-1n9t, C18-2n6c, C18-3n3, C18-3n6, n-3 PUFAs, and n-6 PUFAs in the longissimus dorsi of the TP6 group (*p* < 0.05), and the ratio of n6/n3 in the biceps femoris of lambs changed significantly (*p* < 0.05). In addition, the relative expression levels of myosin heavy chainsI (MyHCI) and MyHCIIa mRNAs in the muscles were significantly increased, and the relative expression levels of MyHCIIX and MyHCIIB mRNAs in the muscles were significantly decreased. And in terms of comprehensive economic benefits, it is most appropriate to add 6 g/kg tea polyphenols to the diet.

## Simple Summary

With intensified farming, livestock grow faster and reach the market sooner, yet meat quality suffers and antibiotic misuse is common. Tea polyphenols, with antioxidant, antibacterial, and anti-inflammatory effects, are promising natural additives. Weaning stress in lambs disrupts growth and metabolism, induces oxidative stress, and impairs muscle development and meat quality. The purpose of this study was to explore the effects of tea polyphenols on the muscle quality, antioxidant capacity, and fatty acid composition of weaned lambs. The results showed that adding 6 g/kg tea polyphenols to the diet was the most appropriate. We hope to provide a theoretical basis for the application of tea polyphenols in the production of young ruminants.

## 1. Introduction

Tea has a long history of utilization and is widely beloved around the world. Tea contains various chemical substances, such as polyphenols, methylxanthines, caffeine, amino acids, etc. As a safe and highly effective natural antioxidant [1,2], tea polyphenols are flavonoid compounds with the basic structure of α-phenyl benzopyran, and their antioxidant function is determined by their special molecular structure [3,4]. At present, a variety of chemical components have been isolated from tea polyphenols, mainly including catechins, flavonoids, anthocyanins, anthocyanins, anocyanins, phenolic acids, and phenolic acid compounds. Among them, catechins account for more than 70% of the total amount of tea polyphenols, consisting of epigallocatechin-3-gallate (L-EGCG), epigallocatechin (L-EGC), epicatechin-3-gallate (L-ECG), and epicatechin (L-EC) [5].

Tea polyphenols, as a natural feed additive of plants, are safer and more efficient compared with synthetic antioxidants [6]. Meanwhile, tea polyphenols can improve the growth performance of livestock and poultry, alleviate stress, and improve meat quality [7,8,9]. An experimental study investigating the inclusion of 24 g/kg green tea by-products in finishing pig diets revealed that this supplementation level neither compromised animal growth performance nor reduced carcass fat deposition while increasing the lean meat percentage. In terms of muscle quality, factors such as the pH value, shear force, and cooked meat rate reflect the physical properties of the meat. Notably, progressive increases in green tea by-product supplementation were correlated with significantly decreased pork shear force (indicating improved meat tenderness), accompanied by beneficial modulations in cholesterol metabolism and fatty acid composition profiles [10]. The composition of fatty acids, such as saturated fatty acids (SFAs), unsaturated fatty acids (MUFAs and PUFAs), and the ratio of n-3/n-6 PUFAs, can reflect the nutritional characteristics of meat quality. In addition, research has shown that tea polyphenols exhibit significant antioxidant capabilities, effectively protecting cells from oxidative damage and maintaining normal mitochondrial function [11]. Xu et al. [12] showed in their study that adding 4–6 g/kg tea polyphenols to the diet had optimal effects, which could enhance the immunity and antioxidant capacity of lambs, inhibit intestinal inflammation and oxidative stress, and reduce cell apoptosis. Xie et al. reported using porcine intestinal epithelial (IPEC-J2) cells as a model system revealed the protective mechanism of tea polyphenols against fluoride-induced oxidative stress. Experimental results indicated that tea polyphenols alleviate cellular damage by effectively inhibiting the generation of reactive oxygen species and maintaining the stability of the mitochondrial membrane potential [13]. In recent years, studies by numerous scholars have shown that the addition of tea polyphenols can significantly improve muscle quality, including enhancing tenderness, increasing the antioxidant capacity, optimizing the fatty acid composition, and improving the formation of flavor compounds. 

Mutton is characterized by high protein, low fat, and rich contents of B vitamins, iron, zinc and other minerals. It also features a tender and juicy texture, unique flavor compounds, optimized fatty acid composition, and high nutritional value. However, weaned lambs face stress responses, digestive disorders, and weakened immune systems during the weaning stage. These factors not only affect the growth performance of lambs but may also have adverse effects on their meat quality. Meat quality traits such as tenderness, color, and antioxidant capacity are fundamentally related to the muscle fiber type composition. Myosin heavy chain (MyHC) isoforms (MyHCI, IIa, IIx, and IIb) are molecular markers that define different types of skeletal muscle fibers, and the relative abundance of these muscle fiber types significantly affects meat quality traits. In addition, the transcriptional coactivator peroxisome proliferator-activated receptor γ coactivator 1α (PGC-1α) is a key regulator of mitochondrial biogenesis and oxidative metabolism, which can modulate the expression of downstream genes [14].

Therefore, in this experiment, tea polyphenols were used as a feed additive, with weaned lambs as the experimental animals, to study the effects of tea polyphenols on the muscle quality, antioxidant capacity, and muscle fatty acid composition of weaned lambs, as well as to explore the impact of tea polyphenols on the expression levels of relevant genes in the PGC-1α pathway. The purpose is to provide a theoretical basis for the application of tea polyphenols in the production of young ruminants.

## 2. Materials and Methods

### 2.1. Animals and Experimental Protocol

The tea polyphenols used in this study were provided by Xi’an Best Biotechnology Co., Ltd. (Xi’an, China). The tea polyphenols are in the form of a tea-brown powder, with a special odor. The purity is 98.1%, the content of EGCG is 54.2%, the level of catechins is 86.6%, and the concentration of caffeine is 0.4%.

This experiment was carried out from April 2023 to July 2023 at the Zhuangyuan Black Goat Breeding Co., Ltd. in Leizhou City, China. Forty 45-day-old weaned Leizhou goat lambs with similar body weights (9.1 ± 0.8 kg) and in good health were selected for the experiment. The experiment included a 7-day pre-feeding period and a 45-day formal feeding period. The lambs were randomly divided into 4 treatment groups, with 5 replicates in each group and 2 lambs in each replicate. The control group (CON) was fed a basal diet, while the other three groups were, respectively, supplemented with 2, 4 and 6 g/kg of tea polyphenols in the basal diet, which were denoted as the TP2, TP4, and TP6 groups, respectively. The tea polyphenols to be fed daily were evenly mixed into the concentrate and fed twice a day at 08:00 and 17:00, the lambs had free access to water, and the formulation of the basal diet (Table 1) was in accordance with the nutritional requirements of the Feeding Standard of Goats, China.

### 2.2. Sample Collection

After the experiment, 6 lambs were randomly selected from each group and slaughtered by carotid artery bleeding. The lambs were fasted and deprived of water for 12 h before slaughter. After slaughter, the longissimus dorsi muscle, biceps femoris muscle, and triceps brachii muscle were taken. These muscles were stored at 4 °C for 24 h, and then the meat quality indexes were determined. After that, samples of approximately 3 cm from the longissimus dorsi muscle, biceps femoris muscle, and triceps brachii muscle were taken and placed in 2 cm enzyme-free tubes and RNA tubes. The samples were numbered, quickly frozen in liquid nitrogen, and then transferred to −80 °C for storage, which were used for subsequent RT-qPCR detection.

### 2.3. Determination of Meat Quality

The pH of the muscle was measured at 45 min and 24 h after slaughter. It was measured with a portable pH meter. First, the value at 45 min after slaughter was measured (recorded as pH_0h_), and then the serum was stored in a refrigerator at 4 °C for 24 h before measuring its pH again (recorded as pH_24h_).

Muscle samples were collected within 1 to 2 h after slaughter for measurement. Using a strain-type vertical lateral limit compression instrument, a pressure of 30 kg was applied to the meat sample, and it was weighed after maintaining the pressure for 5 min. The formula for calculating the water loss rate is Water loss rate = (Weight of the meat sample before compression − Weight of the meat sample after compression)/Weight of the meat sample before compression × 100%. The water loss rate reflects the amount of water lost by the meat sample after slaughter.

Muscle samples were collected within 1 to 2 h after slaughter for measurement. The meat sample was put into an aluminum pot and steamed for 45 min. After taking it out, it was cooled for 30 min and then weighed. The formula for calculating the cooked meat rate is Cooked meat rate = Weight of the cooked meat sample/Weight of the raw meat sample × 100%. The cooked meat rate indicates the changes in the meat sample during the steaming process.

After the stripped muscle sample was stored for 24 h, it was heated in a water bath at a constant temperature of 80 °C until the central temperature reached about 70 °C. Then the sample was taken out, the fascia and visible surface fat were removed, and samples were taken in a direction perpendicular to the muscle fibers. The shear value of the muscle was measured with a C-LM type tenderometer. The shear force reflects the tenderness and texture of the muscle.

### 2.4. Determination of Antioxidant Indicators

The activities of total superoxide dismutase (T-SOD) (Catalog No. A001-1-2), glutathione peroxidase (GSH-Px) (Catalog No. A005-1-2), and catalase (CAT) (Catalog No. A007-1-1), as well as the contents of the total antioxidant capacity (T-AOC) (Catalog No. A015-2-1), hydrogen peroxide (H_2_O_2_) (Catalog No. A064-1-1), and malondialdehyde (MDA) (Catalog No. A003-1-2), were measured using reagent kits from Nanjing Jiancheng Bioengineering Institute. The operations were performed in strict accordance with the instructions of the reagent kits.

### 2.5. Determination of Fatty Acid Indexes

Take out the biological samples stored at ultra-low temperature and grind them with a grinder (30 Hz, 1 min) until they turn into powder. Then, weigh 50 mg of the sample into a new EP tube. Add 150 μL of a methanol solution, 200 μL of a methyl tert-butyl ether solution, and 50 μL of a 36% phosphoric acid solution for extraction. Vortex for 3 min. After centrifuging at 4 °C and 14,000× *g* for 5 min, transfer 200 μL of the supernatant to a nitrogen blowing instrument and blow it dry. Add 300 μL of a 15% boron trifluoride–methanol solution, and vortex for 3 min. Keep it in an oven at 60 °C for 30 min. Cool it to room temperature, accurately add 500 μL of an n-hexane solution and 200 μL of a saturated sodium chloride solution, and vortex for 3 min. After centrifuging at 4 °C and 14,000× *g* for 5 min, transfer 100 μL of the n-hexane layer for analysis using a chromatographic analyzer. The detection was carried out by Wuhan Servicebio Technology Co., Ltd. (Wuhan, China).

### 2.6. RT-qPCR

The methods for extracting RNA from the longissimus dorsi muscle, biceps femoris muscle, and triceps brachii muscle, reverse transcribing it into cDNA, and performing real-time quantitative polymerase chain reaction (qPCR) analysis were carried out. The primers were designed by Primer Blast in NCBI (Table 2), and then passed on to Shenggong Biotechnology Co., Ltd. (Shanghai, China) for synthesis. According to the instructions of ChamQ Universal SYBR qPCR Master Mix (Vazyme Biotech Co., Ltd., Nanjing, China), the expression levels of the β-actin and GAPDH mRNAs were used for normalization and calculation of the results, and the results were calculated using the 2^ΔΔCt^ method.

### 2.7. Statistical Analysis

The experimental data were first sorted and calculated in Excel 2019. Subsequently, a one-way analysis of variance (ANOVA) procedure in the SPSS 26.0 software were used for the analysis. Duncan’s multiple range test was employed for multiple comparisons. The final results are presented as the mean values and the standard errors of the means (SEMs). A value of *p* < 0.05 indicates a significant difference.

## 3. Results

### 3.1. Effect of Tea Polyphenols on the Muscle Quality of Weaned Lambs

As presented in Table 3, compared with the CON group, the addition of tea polyphenols in the diet significantly increased (*p* < 0.05) the cooked meat rate of the biceps femoris muscle in the TP4 and TP6 groups, while there was no significant difference between the TP2 group and the CON group (*p* > 0.05). Additionally, it significantly increased (*p* < 0.05) the pH_0h_ and pH_24h_ values of arm triceps in the TP6 group, with no significant differences observed in the pH_0h_ and pH_24h_ values of arm triceps between the TP2 and TP4 groups and the CON group (*p* > 0.05).

### 3.2. Effects of Tea Polyphenols on the Antioxidant Properties of Weaned Lambs’ Muscles 

As shown in Table 4, compared with the CON group, dietary supplementation of tea polyphenols significantly increased the activity of T-SOD in the longissimus dorsi muscle (*p* < 0.05). The TP6 group showed significantly increased activity of GSH-Px (*p* < 0.05) and a significantly reduced level of H_2_O_2_ (*p* < 0.05), while the TP4 group showed significantly improved activity of CAT. There were no significant differences in the activity of GSH-Px and the content of H_2_O_2_ between the TP2 and TP4 groups and the CON group (*p* > 0.05), and no significant differences in the activity of CAT were observed between the TP2 and TP6 groups and the CON group (*p* > 0.05).

In the biceps femoris muscle, compared with the CON group, dietary supplementation of tea polyphenols significantly increased GSH-Px activity (*p* < 0.05) and significantly reduced the H_2_O_2_ content (*p* <0.05). Compared with the CON group, the TP4 and TP6 groups showed a significantly higher T-AOC content (*p* <0.05) and significantly lower MDA content (*p* < 0.05), while there was no significant difference in these indices between the TP2 group and the CON group (*p* > 0.05). The TP6 group exhibited a significant increase in T-SOD activity (*p* <0.05); the TP4 group showed a significant increase in CAT activity (*p* < 0.05).

In the arm triceps muscle, compared with the CON group, dietary supplementation of tea polyphenols significantly increased CAT activity in the TP2 and TP6 groups (*p* < 0.05), while the content of H_2_O_2_ was significantly reduced (*p* < 0.05). However, there were no significant changes in the H_2_O_2_ content and CAT activity in the TP4 group (*p* > 0.05). Additionally, compared with the CON group, the TP4 and TP6 groups showed significantly higher GSH-Px activity (*p* < 0.05), and the TP6 group exhibited a significant increase in T-SOD activity (*p* < 0.05). No significant changes were observed in these indices in the other groups (*p* > 0.05).

### 3.3. Effects of Tea Polyphenols on the Fatty Acids in the Muscles of Weaned Lambs

As can be seen in Table 5, compared with the CON group, the addition of tea polyphenols to the diet significantly increased (*p* < 0.05) the contents of C16-1, C18-1n9t, C18-2n6c, C18-3n3, C18-3n6, n-3 PUFAs, and n-6 PUFAs in the longissimus dorsi muscle of lambs in the TP6 group, there were no significant differences between the TP2 and TP4 groups (*p* > 0.05), and the contents of MUFAs in the muscles of lambs in the TP4 and TP6 groups were significantly increased (*p* < 0.05), while there was no significant difference in the TP2 group (*p* > 0.05). As can be seen in Table 6, compared with the CON group, there were significant changes (*p* < 0.05) in the n6/n3 ratio in the biceps femoris muscle of lambs. As can be seen in Table 7, compared with the CON group, the addition of tea polyphenols to the diet significantly decreased (*p* < 0.05) the content of C17-0 in the triceps brachii muscle of lambs, and the contents of C20-1(cis-11) in the TP4 and TP6 groups were significantly decreased (*p* < 0.05), but there were no significant differences in the TP2 group (*p* > 0.05).

### 3.4. Effects of Tea Polyphenols on the Relative Expression Level of the MyHC mRNA in Weaned Lamb Muscles

**Figure 1:**
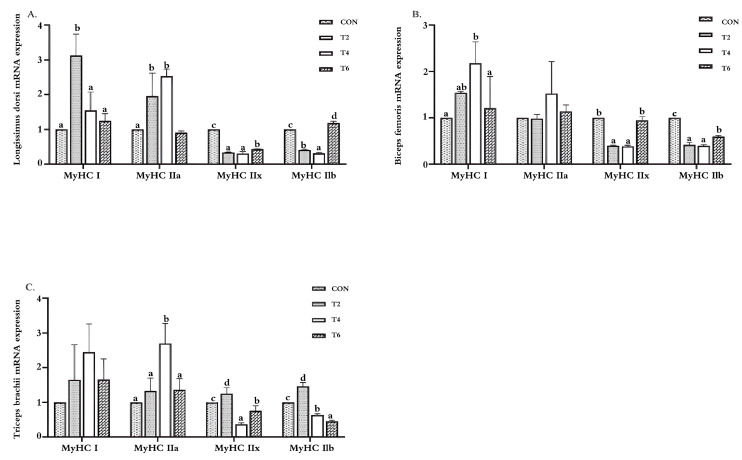
Relative expression of the MyHC mRNA in lamb muscles. CON, basal diet; TP2, basal diet + 2 g/kg tea polyphenols; TP4, basal diet + 4 g/kg tea polyphenols; TP6, basal diet + 6 g/kg tea polyphenols. (**A**–**C**) The relative expression levels of MyHCI, MyHCIIa, MyHCIIx, and MyHCIIb in the longissimus dorsi, biceps femoris, and triceps brachii muscles, respectively. (**A**) Longissimus dorsi muscle; (**B**) biceps femoris muscle; (**C**) triceps brachii muscle. ^a–d^ Values in the same row with different letters are significantly different (*p* < 0.05). The results are presented as the means ± SEMs (*n* = 10).

As can be seen in Figure 1, compared with the CON group, the addition of tea polyphenols to the feed significantly decreased (*p* < 0.05) the relative expression levels of the MyHCIIx mRNA in the longissimus dorsi muscle and MyHCIIb mRNA in the biceps femoris muscle. Compared with the CON group, the TP2 and TP4 groups showed significantly increased (*p* < 0.05) relative expression levels of the MyHCIIa mRNA in the longissimus dorsi muscle, there was no significant change in the TP6 group (*p* > 0.05), and the TP2 and TP4 groups showed significantly decreased (*p* < 0.05) relative expression levels of the MyHCIIb mRNA. Compared with the CON group, the TP4 and TP6 groups showed significantly decreased (*p* < 0.05) relative expression levels of the MyHCIIx mRNA in the triceps brachii muscle, and there were significant decreases (*p* < 0.05) in the biceps femoris of the TP2 and TP4 groups; the TP4 and TP6 groups presented significantly decreased (*p* < 0.05) relative expression levels of the MyHCIIb mRNA in the triceps brachii muscle. Compared with the CON group, the TP2 group showed a significant increase (*p* < 0.05) in the relative expression level of the MyHCI mRNA in the longissimus dorsi muscle. This group also presented a significant increase (*p* < 0.05) in the relative expression levels of the MyHCIIx and MyHCIIb mRNAs in the triceps brachii muscle. Compared with the CON group, the TP4 group presented a significant increase in the relative expression level of the MyHCI mRNA in the biceps femoris muscle (*p* < 0.05), while there were no significant differences in the TP2 and TP6 groups (*p* > 0.05).

### 3.5. Effect of Tea Polyphenols on the Expression of Related Genes in the PGC-α Pathway in the Longissimus Dorsi Muscle

**Figure 2:**
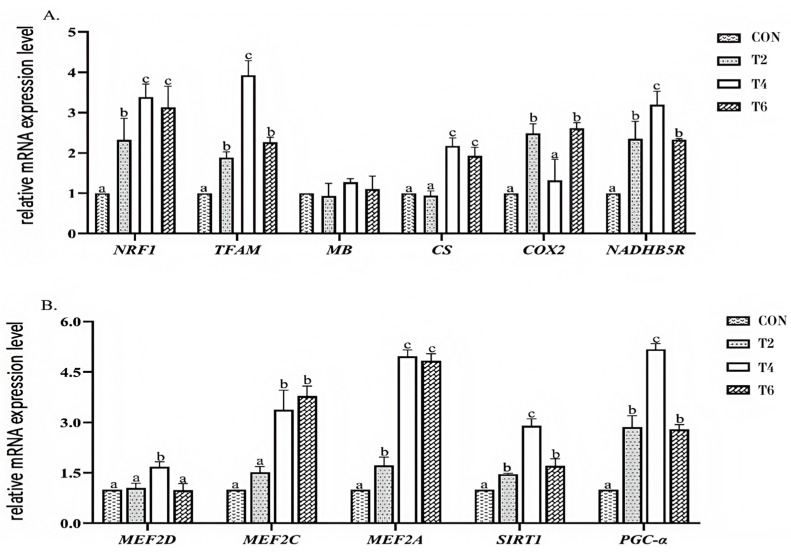
Relative expression of PGC-α pathway- and mitochondria-related mRNAs in the longissimus dorsi muscle (**A**,**B**). CON, basal diet; TP2, basal diet + 2 g/kg tea polyphenols; TP4, basal diet + 4 g/kg tea polyphenols; TP6, basal diet + 6 g/kg tea polyphenols. NRF1, Nuclar respiratory factor-1; TFAM, Transcription Factor A Mitochondrial; MB, Myoglobin; CS, Citrate-synthase; COX2, Cyclooxygenase-2; NADHB5R, Cytochrome b5 reductase; MEF2D, Myocyte enhancer factor 2D; MEF2C, Myocyte enhancer factor 2C; MEF2A, Myocyte enhancer factor 2A; SIRT1, sirtuin-1;PGC-1α, Peroxisome proliIerators-activated receptor γ coactivator α. ^a–c^ Values in the same row with different letters are significantly different (*p* < 0.05). The results are presented as the means ± SEMs (*n* = 10).

The longissimus dorsi muscle, the most representative muscle, was selected to explore the underlying mechanism of tea polyphenols. As shown in Figure 2, compared with the CON group, tea polyphenols significantly increased (*p* < 0.05) the relative expression levels of the NRF1, TFAM, NADHB5R, SIRT1, PGC-α, and MEF2A mRNAs in the longissimus dorsi muscle. Additionally, compared with the CON group, the TP4 and TP6 groups exhibited significantly increased (*p* < 0.05) relative expression levels of the CS and MEF2C mRNAs, but there were no significant differences in the TP2 group (*p* > 0.05). The TP2 and TP6 groups showed significant increases (*p* < 0.05) in the relative expression level of the COX2 mRNA in the longissimus dorsi muscle. Moreover, the TP4 group also presented a significant increase (*p* < 0.05) in the relative expression level of the MEF2D mRNA in the longissimus dorsi muscle, while there were no significant differences in the TP2 and TP6 groups (*p* > 0.05). 

## 4. Discussion

### 4.1. Effect of Tea Polyphenol Administration on the Muscle Quality of Weaned Lambs 

Meat quality refers to the physical and chemical properties related to the appearance, palatability, and nutritional value of fresh or processed meat, directly reflecting its overall quality [21]. After the slaughter of livestock and poultry, anaerobic glycolysis of muscle glycogen produces lactic acid. Since lactic acid fails to be discharged from the muscle, a decrease in muscle pH occurs. Therefore, pH serves as an indicator of the rate and extent of postmortem glycolysis. A reduction in pH not only affects the muscle’s water-holding capacity and tenderness [22] but also accelerates protein degradation, influencing parameters such as shear force and cooking yield. Thus, pH is a critical factor in evaluating meat quality [23]. Research has shown that tea polyphenols can not only improve the flavor and color of meat but also inhibit fat oxidation, thereby extending the shelf life of meat and enhancing its nutritional value [24]. The results of this study showed that adding 4 g/kg and 6 g/kg of tea polyphenols to the diet could significantly increase the cooked meat rate of the biceps femoris muscle; the level of cooked meat yield directly reflects the water-holding capacity during the thermal denaturation of muscle proteins—this property is crucial for the juiciness and tenderness of meat, and its essence lies in the difference in the ability of muscles to retain moisture during heating. Adding 6 g/kg of tea polyphenols could significantly increase the pH values of the triceps brachii muscle at 0 h and 24 h. This indicates that the tea polyphenol intervention effectively slows down the acidification process of muscles in the early postmortem period. A higher pH value is usually associated with a better muscle water-holding capacity, lower cooking loss, and better tenderness, and serves as one of the important indicators for improving meat quality. Consistent with our findings, Zhong et al. [25] reported that dietary supplementation with tea polyphenols in parent pigeons significantly increased the 24-h postmortem pH of squab muscle, while reducing the muscle shear force and moisture content, thereby enhancing meat quality. Similarly, Zhong et al. [26] demonstrated that dietary tea polyphenol supplementation significantly altered the 24 h postmortem pH of the gluteus medius and psoas major muscles in goats after 40 and 60 days. They also showed that tea polyphenols enhanced the total antioxidant status, suppressed lipid oxidation, increased intramuscular fat deposition, and elevated the total heme content in goat muscle. Moreover, Gao et al. found that tea polyphenols improved the myofibril fragmentation index and muscle microstructure in yak meat, underscoring their potential to enhance meat tenderness for the food industry [27]. Previous studies have confirmed that tea polyphenols can extend the shelf life and enhance nutritional value of meat by improving its flavor and color, inhibiting fat oxidation, and, at the same time, they can also reduce the generation of unpleasant odors such as aldehydes, further strengthening the stability of meat quality. Combined with the results of this study, these findings collectively confirm that tea polyphenols can improve meat quality in multiple dimensions, including the water-holding capacity, tenderness, and color, by regulating the physiological and biochemical processes in postmortem muscle. This provides a theoretical basis for their application as natural meat quality improvers.

### 4.2. Effect of Tea Polyphenols on the Antioxidant Activity of Weaned Lamb Muscles

After the slaughter of livestock and poultry, the loss of the muscles’ antioxidant system, along with the generation of reactive oxygen species, cellular damage, and exposure to heme and non-heme iron as well as light, will cause severe lipid peroxidation and protein oxidation. These oxidation processes are one of the core mechanisms leading to the decline in meat quality. Fat oxidation and rancidity produce unpleasant flavors and odors; protein denaturation leads to a decrease in the water-holding capacity, increased juice loss, and reduced tenderness; and the oxidation of myoglobin to ferric myoglobin causes changes in meat color [28]. Therefore, maintaining or enhancing the antioxidant capacity of the muscle after slaughter is the key to improving and preserving the quality of the meat. GSH-Px is an antioxidant present in mammals and plays an important role in a variety of detoxification reactions and the inhibition of lipid peroxidation reactions [29]. The liver is the main site of glutathione metabolism. Glutathione and its related enzymes form an antioxidant defense system that can prevent the occurrence of oxidative stress. CAT is an important enzyme, and it usually has an inverse relationship with H_2_O_2_. It is responsible for removing H_2_O_2_ produced under various stress conditions and protecting cells from damage caused by oxidative stress. SOD plays an important role in protecting cells from the toxic effects of superoxide radicals by catalyzing the transformation reaction of free radicals. As mentioned before, GSH-Px, CAT, and SOD constitute the initial defense against ROS, and decreases in their activities contribute to the occurrence of oxidative damage in tissues [30]. Malondialdehyde (MDA), the final product of lipid oxidation, can reflect the degree of oxidation in the body. Its accumulation can lead to the rancidity of muscle fatty acids and the deterioration of flavor. However, tea polyphenols delay the oxidation and rancidity of meat by reducing the production of MDA. The results of this study showed that tea polyphenols significantly increased the activities of T-SOD, GSH-Px, and CAT in the muscles of lambs, and significantly reduced the content of H_2_O_2_. In addition, the T-AOC in the biceps femoris muscle of lambs was significantly increased, and the content of MDA was significantly decreased. These indicators indicate that tea polyphenols effectively enhance the antioxidant capacity of muscle tissue and significantly reduce the oxidative damage suffered by muscle post-slaughter. This effective inhibition of oxidative damage is crucial for improving and maintaining meat quality. Therefore, tea polyphenols effectively reduce the decline in meat quality caused by oxidative damage by enhancing the antioxidant capacity of the muscle itself. The results show that in lamb meat with added tea polyphenols, the level of the oxidative stress marker MDA was significantly reduced, while the antioxidant capacity of the lamb was also significantly enhanced. This finding indicates that tea polyphenols play a crucial role in alleviating oxidative stress during the growth of lambs, thereby potentially supporting enhanced growth performance and meat quality. This further emphasizes the value of tea polyphenols in improving the economic benefits of meat sheep farming [31]. In addition, the inhibitory effects of tea polyphenols on the formation of heterocyclic aromatic amines (HAAs) in grilled lamb patties were evaluated at two cooking temperatures (220 °C and 250 °C). The results showed that at 220 °C, tea polyphenols at a level of 0.5% significantly reduced HAA formation, demonstrating strong antioxidant properties [32], which further expands its value in improving meat quality—not only enhancing the quality of fresh meat but also reducing the generation of potential harmful substances during processing and strengthening the safety of meat consumption. In this study, the addition of 6 g/kg tea polyphenols significantly enhanced the antioxidant capacity in lamb muscle, increased the activities of antioxidant-related enzymes in the body, enhanced resistance to lipid oxidation, and reduced the levels of MDA and H_2_O_2_; these findings confirm its crucial role as a natural antioxidant in regulating meat quality.

### 4.3. Effects of Tea Polyphenols on Fatty Acids in the Muscles of Weaned Lambs

Fatty acids in meat are essential components that enhance muscle nutritional value by supporting key physiological and biochemical functions [33]. The relative composition and abundance of fatty acids critically shape ruminant meat attributes—flavor, texture, tenderness and nutritional profile. As essential nutrients, they not only support animal health but also govern meat quality, directly influencing consumer preferences and market demand [34]. In the muscle fat of ruminants, the content of SFAs is relatively high, while the content of PUFAs that are beneficial for health is low. Consuming long-chain n-3 fatty acids such as eicosapentaenoic acid and docosahexaenoic acid brings many benefits to human health [35]. However, the consumption of SFAs has raised concerns about their potential to increase the risks of cardiovascular diseases and metabolic syndrome. Therefore, the meat quality of ruminants is considered less healthy compared to that of monogastric animals. Therefore, controlling the fatty acid composition in meat to reduce the content of SFAs and the n-6/n-3 ratio so as to improve the levels of PUFAs in meat has become the main objective in the research field of ruminants [36]. Some studies have shown that the content of fatty acids is strongly influenced by nutritional factors [37]. This study shows that adding 6 g/kg tea polyphenols to the diet significantly increases the contents of C16-1, C18-1n9t, C18-2n6c, C18-3n3, C18-3n6, MUFAs, n-3 PUFAs, and n-6 PUFAs in the longissimus dorsi muscle of lambs, and the n6/n3 ratio in the biceps femoris muscle of lambs decreases significantly. Optimizing the fatty acid composition helps form better flavor characteristics. Meanwhile, this study also confirms that tea polyphenols significantly enhance the muscle’s antioxidant capacity, which is crucial for protecting beneficial yet more oxidizable unsaturated fatty acids and preventing their oxidative rancidity. The improvements in the antioxidant capacity and fatty acid composition complement each other, jointly ensuring meat quality. The study by Li Hua et al. [38] showed that supplementation of tea polyphenols in the drinking water of broilers under heat stress significantly increased daily weight gain, as well as the ratios of C18:2 and PUFAs/SFAs, compared with the blank control group. These findings indicate that tea polyphenols can effectively alleviate the adverse impact of heat stress on broilers. Similarly, one study fed goats dietary tea catechins and showed that adding a moderate dose of TC increased the proportion of UFAs in muscle [39]. In summary, this study indicates that dietary supplementation with tea polyphenols can increase the fatty acid content in lamb muscle, elevate the content of polyunsaturated fatty acids (PUFAs), and effectively improve the n6/n3 ratio. This not only significantly enhances the nutritional value and health attributes of lamb meat, making it more in line with modern consumption trends, but also meets consumers’ dual demands for flavor and health, while boosting the market competitiveness of the product. These findings provide an important theoretical basis for the high-quality and efficient development of meat sheep breeding.

### 4.4. Effects of Tea Polyphenols on the Relative Expression of the MyHC mRNA in the Muscle of Weaned Lambs

Skeletal muscle is the most abundant tissue in mammals, characterized by contraction and extension [40], and is composed of different types of muscle fibers. Its physical and chemical properties are closely related to the muscle quality of animals. The type of muscle fiber has different effects on the meat quality of livestock and poultry [41]. Mammalian skeletal muscle fibers can be classified into slow oxidative type I, fast oxidative type IIa, intermediate type IIx, and fast glycolytic type IIb [42], and are distinguished according to the expression of myosin heavy chain subtypes [43]. Type I and IIa fibers are predominantly engaged in oxidative metabolism and exhibit strong fatigue resistance, with their proportion in muscles determining the freshness of meat color and tenderness. Type IIx and IIb fibers are primarily glycolytic and show lower fatigue resistance [44].

Slow myosin heavy chain (MyHC), myoglobin, and troponin I-SS, markers of slow-twitch fiber types, are highly expressed in oxidative muscle fibers [45,46]. Hwang et al. investigated an electronic tongue to study porcine muscle fibers and flavor, and confirmed that there is a positive correlation between the proportion of type IIb fibers and sourness and astringency, and a negative correlation with saltiness. Meanwhile, with the increase in the contents of type I and type IIa fibers in porcine muscle, the umami taste will increase accordingly [47]. Judging from the results of this experiment, adding tea polyphenols to the feed significantly increased the relative expression levels of the MyHCIand MyHCIIa mRNAs in lamb muscles, and significantly decreased the relative expression levels of the MyHCIIx and MyHCIIb mRNAs. The transformation of such muscle fiber types plays a crucial role in improving lamb meat quality, coupled with its positive regulation of the muscle antioxidant capacity and fatty acid composition. Similarly, many natural plant extracts, such as catechins and resveratrol, have been proven to not only regulate the types of muscle fibers [48,49] but also enhance the body’s anti-fatigue ability [50]. However, this study demonstrates that in terms of lamb meat quality, tea polyphenols can achieve this transformation through the targeted regulation of MyHC isoform expression. This provides evidence at the molecular mechanism level for tea polyphenols as a natural meat quality improver, and offers a robust nutritional strategy for the production of high-quality lamb meat.

### 4.5. Effects of Tea Polyphenols on the Expression Levels of Genes Related to the PGC-α Pathway in the Longissimus Dorsi Muscle

The transcriptional coactivator PGC-1α is a nuclear receptor coactivator in the key signaling pathway of muscle fiber type conversion. It is regulated by cell signaling cascades [51]. The PGC-1α gene may influence muscle fiber conversion through multiple pathways, inducing the transformation of type IIx muscle fibers to type I fibers with directionality [52]. Originally identified as a protein interacting with PPARγ, PGC-1α expression increases in skeletal muscle and brown adipose tissue in cold environments [53]. Notably, studies have shown that muscle-specific overexpression of PGC-1α in mice significantly enhances thigh muscle coloration, accompanied by the upregulation of cytochrome c and myoglobin expression, further highlighting PGC-1α’s influence on skeletal muscle coloration and quality characteristics [54].

PGC-1α collaborates with the downstream factors NRF1 and TFAM to regulate mitochondrial biogenesis and reduce mitochondrial ROS production [55], effectively preventing oxidative damage to the key components of post-slaughter muscle, which is crucial for maintaining meat color stability and delaying rancidity. It also synergizes with MEF2 to activate mitochondrial biogenesis and muscle fiber development. Mitochondria play a crucial role in the redox reaction of myoglobin. Additionally, mitochondrial NADH-cytochrome b5 reductase directly influences myoglobin reduction activity by participating in the conversion of metmyoglobin to its reduced form [56,57]. The efficiency of this restoration system is one of the primary factors determining the shelf life, appearance, and consumer acceptance of meat products.

From the results of this experiment, adding 4 and 6 g/kg tea polyphenols to the feed can upregulate the relative expression levels of the NRF1, TFAM, NADHB5R, SIRT1, PGC-α, MEF2, CS, and COX2 mRNAs in the longissimus dorsi muscle of lambs to varying degrees. As downstream effectors of PGC-1α, NRF1, TFAM, and SIRT1 can regulate the replication and transcription of mitochondrial DNA to different extents, thereby driving the coordinated expression of mitochondrial genes [58]. In addition, both TFAM and MEF2 can regulate the transformation among muscle fiber types I↔IIa↔IIx↔IIb. CS and COX2 are marker enzymes reflecting the oxidative capacity of tissues and are located in the inner mitochondrial membrane. The levels of their activities determine the level of mitochondrial biogenesis [59]. NADHB5R exists in mitochondria and can directly participate in the reduction of metmyoglobin, affecting the reduction activity of myoglobin. Therefore, tea polyphenols significantly enhance the antioxidant capacity of muscles by strengthening mitochondrial biosynthesis and antioxidant defense, protecting lipids and proteins from oxidative damage, and ensuring flavor, water-holding capacity, and shelf life.

In conclusion, tea polyphenols can regulate downstream effectors through the SIRT1/PGC-α pathway. This not only promotes the transformation of fast muscle fibers into slow muscle fibers but also enhances the replication, transcription, and biogenesis of mitochondria in muscles. Furthermore, it can increase the rate of the metmyoglobin reduction system (NADHB5R) within the muscles. Ultimately, it can improve meat quality and enhance the color of the muscles.

## 5. Conclusions

In conclusion, these research results show that adding 6 g/kg of tea polyphenols to the diet is most appropriate. It can not only enhance the antioxidant performance of lamb muscles but also improve the muscle quality and increase the concentrations of C16:1, C18:1n9t, C18:2n6c, C18:3n3, C18:3n6, MUFAs, n-3 PUFAs, and n-6 PUFAs in the longissimus dorsi muscle of lambs. These research findings provide a theoretical basis for the development of tea polyphenols as a feed additive to improve the quality of weaned lamb meat.

## Figures and Tables

**Table 1:** Composition and nutrient levels of basal diets (DM basis) %.

Ingredients (%)	Content
Pennisetum	50.00
Corn	29.00
Soybean meal	10.00
Bran	7.50
Premix ^1^	2.00
CaHPO_4_	0.50
NaHCO_3_	0.50
NaCl	0.50
Total	100.00
Nutrient levels	
Dry matter (%) [15]	90.80
Metabolic energy (MJ/Kg) ^2^	10.43
Crude protein [16]	14.69
Crude fat [17]	2.84
Acid detergent fiber [18]	26.23
Neutral detergent fiber [18]	39.9
Ca [19]	0.54
P [20]	1.10

^1^ The premix provided the following per kg of diets: VA 8 000 IU, VD 2 000 IU, VE 40 IU, Cu 12 mg, Fe 70 mg, Mn 50 mg, Zn 80 mg, I 1.0 mg, Se 0.27 mg, and Co 0.3 mg; ^2^ ME was a calculated value, while the others were measured values.

**Table 2:** Nucleotide sequences of the primers used to measure the target genes.

Gene symbol	Product Length, bp	Accession No.	Nucleotide Sequence of Primers (5’→3’)
MyHCI	139	XM_004010325.3	F: CTTCCCCATATATACAGCCCCC R: CTTGGCTTTCAAACGCGCC
MyHCIIα	106	XM_012122422.2	F: AGCTCCAAGGTAAGTGGGAA R: GCCTGGAAGTGAGACGGTTC
MyHCIIx	139	XM_004012706.4	F: TTAAGAAAGAGGGAGGCGAC R: CAAAGACCCTGCCTTGGAGAT
MyHCIIb	182	XM_027974884.1	F: GCCCTTGGAATGAGGCTGAC R: ATGCGCTCCTTTCGGACTT
β-actin	181	XM_001009784.1	F: GCAAATGCTTCTAGGCGGAC R: GGCCATCCCAGCCTCATAAC
COX2	151	1485859	F: AAGACGCAGCATCACCCATTA R: TCTTGTGOGTCTATGGTGCT
CS	142	102168803	F: TCCGTGGCCCAATGTAGATG R: GGGAGAATCCTGGCTCTGAC
MB	292	100860833	F: TTCCCAAATGCTTGACAGGGA R: GAGTTGGGTTTCAGGCCACT
MEF2A	173	102180703	F: AGGGGGAGACTTTGTAGGCA R: CCTGGGGCTGAGGAAAACAT
MEF2C	158	102175290	F: TTAGCCCAGGCTATGGAGGT R: CCCTGAACCACTACTCAGCA
MEF2D	104	102174260	F: ACTGTGCTCATGAACGGTCT R: CGGCTGACAGTCCTCTTGTA
NADHB5R	210	102175455	F: GGAGGGAACACGTGTGCTTA R: TGTAATCTTGATCCAGCACTCAG
NRF1	167	102180186	F: CTGCGCTGTCCGATATCCTG R: GCATCTGGGAACCTATGCCC
PGC-1α	170	100861288	F: ATGGGGCGATCTTGAACGTG R: GGGCTACTCAGTCATGCCAA
SIRT1	155	102180824	F: ATTGGCTGGCAGGAGATACA R: GTGGTCCAACCCACATGACT
TFAM	134	102187195	F: ATGGTTTTAGTCCCAAGTGCC R: AAGCCAACCTGGTTCCAAGA
GAPDH	171	100860872	F: TGAAGGGGTCATTGATGGCA R: TGAAAGAGGGTGAATGGGCA

**Table 3:** Effect of TP administration on the muscle quality of weaned lambs.

Groups						
Item	CON	TP2	TP4	TP6	SEM	*p*-Value
Longissimus dorsi						
pH_0h_	5.61	5.85	5.94	5.90	0.13	0.064
pH_24h_	5.68	5.89	5.81	5.93	0.14	0.299
Water loss rate (%)	15.40	27.30	23.20	26.21	0.06	0.218
Cooked meat rate (%)	68.95	69.03	71.82	62.35	0.03	0.091
Shear force/N	28.68	28.79	28.81	33.21	3.95	0.569
Biceps femoris						
pH_0h_	5.94	6.16	5.72	5.97	0.16	0.081
pH_24h_	5.54	5.70	5.68	5.44	0.11	0.059
Water loss rate (%)	40.30	42.15	38.65	42.85	0.05	0.799
Cooked meat rate (%)	61.28 ^a^	63.13 ^ab^	73.10 ^bc^	76.35 ^c^	0.05	0.019
Shear force/N	26.61	24.73	22.30	29.10	3.57	0.285
Arm triceps						
pH_0h_	5.91 ^a^	5.91 ^a^	6.12 ^ab^	6.25 ^b^	0.14	0.043
pH_24h_	5.55 ^a^	5.57 ^a^	5.60 ^ab^	5.71 ^b^	0.06	0.033
Water loss rate (%)	20.30	26.98	24.58	27.10	0.04	0.291
Cooked meat rate (%)	64.05	62.60	66.08	60.18	0.03	0.215
Shear force/N	29.35	24.45	21.35	27.00	3.30	0.105

In the same row, values with different small superscript letters mean significant differences (*p* < 0.05), while values with the same or no superscript letters mean no significant differences (*p* > 0.05). The same as below. CON, basal diet; TP2, basal diet + 2 g/kg tea polyphenols; TP4, basal diet + 4 g/kg tea polyphenols; TP6, basal diet + 6 g/kg tea polyphenols. SEM, the standard error of the mean. ^a–c^ Values in the same row with different letters are significantly different (*p* < 0.05). The results are presented as the means ± SEMs (*n* = 10).

**Table 4:** Effect of TP administration on muscle antioxidant properties in weaned lambs.

Groups						
Item	CON	TP2	TP4	TP6	SEM	*p*-Value
Longissimus dorsi						
T-SOD (U/mgprot)	24.44 ^a^	36.44 ^b^	36.53 ^b^	36.70 ^b^	3.06	0.001
T-AOC (mmol/gprot)	1.01	1.04	1.00	1.02	0.01	0.131
MDA (nmol/mgprot)	1.28	1.12	1.11	1.17	0.09	0.283
GSH-Px (U/gprot)	19.82 ^a^	25.19 ^ab^	23.73 ^ab^	28.93 ^b^	2.57	0.017
H_2_O_2_ (mmol/gprot)	6.00 ^b^	5.93 ^b^	5.34 ^ab^	4.88 ^a^	0.32	0.006
CAT (U/mgprot)	9.66 ^a^	13.97 ^a^	33.79 ^b^	23.22 ^ab^	6.53	0.026
Biceps femoris						
T-SOD (U/mgprot)	29.66 ^a^	30.23 ^a^	30.37 ^a^	33.59 ^b^	1.04	0.005
T-AOC (mmol/gprot)	0.97 ^a^	0.97 ^a^	1.04 ^b^	1.04 ^b^	0.01	<0.001
MDA (nmol/mgprot)	1.24 ^c^	1.10 ^bc^	0.75 ^a^	1.04 ^b^	0.08	<0.001
GSH-Px (U/gprot)	21.20 ^a^	25.27 ^b^	28.68 ^b^	25.36 ^b^	1.61	0.002
H_2_O_2_ (mmol/gprot)	7.04 ^c^	5.42 ^b^	3.11 ^a^	5.52 ^b^	0.29	<0.001
CAT (U/mgprot)	16.65 ^a^	22.27 ^a^	71.19 ^b^	26.49 ^a^	5.49	<0.001
Arm triceps						
T-SOD (U/mgprot)	44.91 ^a^	50.68 ^a^	47.34 ^a^	60.44 ^b^	3.93	0.004
T-AOC (mmol/gprot)	0.98	0.99	1.02	1.01	0.01	0.59
MDA (nmol/mgprot)	1.17	0.91	0.96	1.04	0.31	0.847
GSH-Px (U/gprot)	34.32 ^a^	39.33 ^ab^	41.10 ^b^	43.92 ^b^	2.58	0.011
H_2_O_2_ (mmol/gprot)	12.03 ^c^	7.44 ^ab^	9.94 ^bc^	6.70 ^a^	1.31	0.002
CAT (U/mgprot)	29.88 ^a^	67.79 ^b^	60.31 ^ab^	81.57 ^b^	15.11	0.047

CON, basal diet; TP2, basal diet + 2 g/kg tea polyphenols; TP4, basal diet + 4 g/kg tea polyphenols; TP6, basal diet + 6 g/kg tea polyphenols; T-SOD, total superoxide dismutase; T-AOC, total antioxidant capacity; MDA, malondialdehyde; GSH-pX, glutathione peroxidase; H_2_O_2_, hydrogen peroxide; CAT, catalase; SEM: the standard error of the mean. ^a–c^ Values in the same row with different letters are significantly different (*p* < 0.05). The results are presented as the means ± SEMs (*n* = 10).

**Table 5:** Effect of TP administration on fatty acids in the longest dorsal muscle of weaned lambs (mg/100 g).

Groups						
Item	CON	TP2	TP4	TP6	SEM	*p*-Value
Decanoic acid C10-0	1.36	1.75	1.38	2.12	0.70	0.670
Lignoceric acid C24-0	5.06	5.03	4.92	4.98	0.07	0.271
Hexanoic acid C6-0	0.17	0.82	0.17	0.16	0.51	0.516
Octanoic acid C8-0	0.42	0.49	0.44	0.54	0.09	0.564
Nonanoic acid C9-0	0.29	0.35	0.34	0.31	0.09	0.889
Myristoleic acid C14-1	2.14	2.87	2.31	3.89	0.85	0.243
Palmitelaidic Acid C16-1T	4.61	4.97	6.65	8.04	1.66	0.219
Linolelaidic acid C18-2n6t	3.01	2.67	3.38	4.48	0.65	0.102
C19:1(Cis-10) acid	5.89	5.35	5.97	6.53	0.37	0.072
Henicosanoic acid C21-0	2.11	2.11	2.06	2.08	0.04	0.372
Cis-4,7,10,13,16,19-docosahexaenoic acid	10.68	13.27	7.15	20.71	4.62	0.089
All-cis-7,10,13,16,19-docosapentaenoic acid	28.47	29.04	25.95	37.37	4.80	0.174
Tricosanoic acid C23-0	4.28	4.29	4.20	4.24	0.06	0.436
Palmitic acid C16-0	490.26	457.31	541.78	704.28	95.75	0.123
Cis-9-palmitoleic acid C16-1	12.21 ^a^	11.77 ^a^	15.22 ^a^	25.75 ^b^	3.82	0.021
Heptadecanoic acid C17-0	11.16	10.37	13.04	14.80	2.82	0.444
Stearic acid C18-0	482.31	390.01	515.24	615.11	79.09	0.112
Oleic acid C18-1n9c	310.04	229.04	414.63	681.48	146.23	0.064
Elaidic acid C18-1n9t	32.87 ^a^	30.48 ^a^	44.55 ^ab^	67.89 ^b^	11.57	0.042
Linoleic acid C18-2n6c	136.10 ^a^	123.26 ^a^	178.87 ^a^	321.44 ^b^	59.66	0.037
A-Linolenic acid C18-3n3	5.20 ^a^	4.62 ^a^	7.13 ^ab^	13.34 ^b^	2.74	0.045
Gamma linolenic acid C18-3n6	5.54 ^a^	5.64 ^a^	5.91 ^a^	7.24 ^b^	0.54	0.047
Nonadecanoic acidC19-0	2.80	2.75	2.72	2.80	0.11	0.861
Arachidonic acid C20-0	3.28	2.80	2.80	3.11	0.43	0.631
Cis-11-eicosenoiccid C20-1(cis-11)	4.48	3.08	3.90	4.52	0.79	0.298
Cis-11,14-eicosadienoic acid C20-2	3.89	3.35	3.84	4.30	0.33	0.107
Cis-8,11,14-eicosatrienoic acid C20-3n6	11.71	12.70	11.94	17.12	2.50	0.184
Arachidonic acid C20-4n6	160.89	165.69	164.90	239.82	43.00	0.272
Eicosapentaenoic acid C20-5n3	27.01	26.41	26.84	38.04	5.90	0.221
Saturated fatty acids (SFAs)	1024.02	905.63	1110.93	1387.1	175.58	0.114
Unsaturated fatty acids (UFAs)	828.72	752.44	1020	1619.26	288.59	0.062
Monounsaturated fatty acids (MUFAs)	364.27 ^a^	281.80 ^a^	485.64 ^b^	789.59 ^c^	161.73	0.049
Polyunsaturated fatty acids (PUFAs)	345.05 ^a^	338.90 ^a^	390.80 ^a^	640.59 ^b^	103.91	0.049
n6/n3	7.02	6.59	8.49	7.91	1.03	0.311
n-3 Polyunsaturated fatty acids (n-3 PUFAs)	42.96 ^a^	44.30 ^a^	41.12 ^a^	72.09 ^b^	10.66	0.048
n-6 Polyunsaturated fatty acids (n-6 PUFAs)	302.54 ^a^	294.59 ^a^	349.68 ^a^	568.50 ^b^	94.82	0.050

CON, basal diet; TP2, basal diet + 2 g/kg tea polyphenols; TP4, basal diet + 4 g/kg tea polyphenols; TP6, basal diet + 6 g/kg tea polyphenols; n6/n3, n-6 polyunsaturated fatty acids/n-3 polyunsaturated fatty acids. SEM: the standard error of the mean. ^a–c^ Values in the same row with different letters are significantly different (*p* < 0.05). The results are presented as the means ± SEMs (*n* = 10).

**Table 6:** Effect of TP administration on fatty acids in the biceps femoris muscle of weaned lambs (mg/100 g).

Groups						
Item	CON	TP2	TP4	TP6	SEM	*p*-Value
Decanoic acid C10-0	1.57	1.13	1.01	1.32	0.44	0.615
Lignoceric acid C24-0	5.04	4.95	5.01	5.02	0.12	0.894
Hexanoic acid C6-0	0.19	0.14	0.08	0.11	0.07	0.526
Octanoic acid C8-0	0.47	0.39	0.41	0.43	0.04	0.227
Nonanoic acid C9-0	0.20	0.22	0.26	0.23	0.04	0.503
Myristoleic acid C14-1	2.81	2.95	2.23	2.69	0.86	0.851
Palmitelaidic Acid C16-1T	6.10	9.63	5.82	7.17	2.24	0.371
Linolelaidic acid C18-2n6t	3.11	3.36	3.49	3.21	0.30	0.635
C19:1(Cis-10) acid	6.11	5.82	6.01	5.79	0.36	0.782
Henicosanoic acid C21-0	2.11	2.07	2.09	2.09	0.05	0.849
Cis-4,7,10,13,16,19-docosahexaenoic acid	8.91	9.52	7.71	12.23	3.46	0.626
All-cis-7,10,13,16,19-docosapentaenoic acid	33.72	30.98	28.43	32.03	7.75	0.918
Tricosanoic acid C23-0	4.31	4.24	4.28	4.29	0.10	0.907
Palmitic acid C16-0	482.01	477.27	488.70	477.08	47.98	0.994
Cis-9-palmitoleic acid C16-1	15.04	16.05	13.80	14.16	3.18	0.894
Heptadecanoic acid C17-0	11.29	11.07	11.54	10.10	1.32	0.975
Stearic acid C18-0	549.43	460.22	483.37	445.46	51.22	0.902
Oleic acid C18-1n9c	357.41	347.92	313.87	328.69	74.45	0.935
Elaidic acid C18-1n9t	44.79	64.99	46.43	48.87	8.91	0.168
Linoleic acid C18-2n6c	170.61	286.40	200.58	181.52	43.56	0.101
A-Linolenic acid C18-3n3	7.03	8.21	6.47	6.89	1.63	0.745
Gamma linolenic acid C18-3n6	5.63	6.47	5.89	6.00	0.39	0.262
Nonadecanoic acidC19-0	2.77	2.74	2.77	2.73	0.09	0.963
Arachidonic acid C20-0	2.73	2.50	2.59	2.54	0.28	0.845
Cis-11-eicosenoiccid C20-1(cis-11)	3.88	3.63	3.72	3.12	0.47	0.442
Cis-11,14-eicosadienoic acid C20-2	3.97	4.23	4.09	3.73	0.28	0.400
Cis-8,11,14-eicosatrienoic acid C20-3n6	12.28	12.86	10.98	12.04	2.52	0.897
Arachidonic acid C20-4n6	178.49	194.53	142.56	139.84	48.34	0.620
Eicosapentaenoic acid C20-5n3	26.50	23.63	24.57	33.08	6.56	0.507
Saturated fatty acids (SFAs)	996.28	991.05	1024.77	976.17	102.10	0.969
Unsaturated fatty acids (UFAs)	973.51	1144.11	896.58	912.55	226.64	0.693
Monounsaturated fatty acids (MUFAs)	428.60	443.44	384.02	403.53	86.94	0.904
Polyunsaturated fatty acids (PUFAs)	397.17	528.76	387.77	379.56	100.12	0.443
n6/n3	8.34 ^ab^	11.65 ^c^	8.98 ^b^	6.61 ^a^	0.89	0.003
n-3 Polyunsaturated fatty acids (n-3 PUFAs)	42.45	41.36	38.74	52.20	11.11	0.655
n-6 Polyunsaturated fatty acids (n-6 PUFAs)	354.73	487.40	349.03	327.36	91.67	0.351

CON, basal diet; TP2, basal diet + 2 g/kg tea polyphenols; TP4, basal diet + 4 g/kg tea polyphenols; TP6, basal diet + 6 g/kg tea polyphenols; n6/n3, n-6 polyunsaturated fatty acids/n-3 polyunsaturated fatty acids. SEM: the standard error of the mean. ^a–c^ Values in the same row with different letters are significantly different (*p* < 0.05). The results are presented as the means ± SEMs (*n* = 10).

**Table 7:** Effect of TP administration on fatty acids in the triceps brachii muscle of weaned lambs (mg/100 g).

Groups						
Item	CON	TP2	TP4	TP6	SEM	*p*-Value
Decanoic acid C10-0	1.45	1.72	1.31	2.13	0.59	0.554
Lignoceric acid C24-0	5.04	4.98	5.06	5.05	0.09	0.771
Hexanoic acid C6-0	0.11	0.11	0.14	0.15	0.05	0.840
Octanoic acid C8-0	0.53	0.39	0.40	0.46	0.08	0.362
Nonanoic acid C9-0	0.26	0.21	0.23	0.30	0.05	0.328
Myristoleic acid C14-1	2.57	2.84	2.44	3.32	0.75	0.666
Palmitelaidic Acid C16-1T	7.57	6.70	6.58	7.98	1.97	0.868
Linolelaidic acid C18-2n6t	4.93	4.38	4.21	4.16	0.68	0.667
C19:1(Cis-10) acid	6.47	6.04	5.70	5.87	0.37	0.265
Henicosanoic acid C21-0	2.12	2.10	2.11	2.10	0.04	0.973
Cis-4,7,10,13,16,19-docosahexaenoic acid	11.29	13.07	7.61	11.71	3.75	0.539
All-cis-7,10,13,16,19-docosapentaenoic acid	39.09	37.02	29.07	27.35	6.59	0.277
Tricosanoic acid C23-0	4.30	4.25	4.32	4.31	0.07	0.786
Palmitic acid C16-0	663.36	471.04	515.80	531.32	92.36	0.263
Cis-9-palmitoleic acid C16-1	18.78	14.13	15.80	18.69	4.68	0.708
Heptadecanoic acid C17-0	15.64 ^b^	11.96 ^a^	11.38 ^a^	11.64 ^a^	1.33	0.038
Stearic acid C18-0	635.21	470.78	499.38	485.00	85.29	0.266
Oleic acid C18-1n9c	581.03	424.06	463.53	431.03	96.56	0.391
Elaidic acid C18-1n9t	70.23	62.06	40.72	61.52	16.30	0.372
Linoleic acid C18-2n6c	326.74	311.04	247.49	239.51	79.31	0.622
A-Linolenic acid C18-3n3	10.97	10.93	9.36	8.23	2.30	0.599
Gamma linolenic acid C18-3n6	6.20	5.87	6.20	6.25	0.48	0.847
Nonadecanoic acidC19-0	2.96	2.83	2.81	2.77	0.10	0.278
Arachidonic acid C20-0	3.07	2.73	2.61	2.51	0.31	0.358
Cis-11-eicosenoiccid C20-1(cis-11)	4.83 ^b^	3.86 ^ab^	3.63 ^a^	3.33 ^a^	0.45	0.048
Cis-11,14-eicosadienoic acid C20-2	4.65	4.36	3.72	3.78	0.42	0.154
Cis-8,11,14-eicosatrienoic acid C20-3n6	16.23	13.69	13.05	12.63	3.17	0.679
Arachidonic acid C20-4n6	236.47	144.44	187.07	130.79	46.59	0.180
Eicosapentaenoic acid C20-5n3	31.63	29.54	26.11	32.05	6.90	0.818
Saturated fatty acids (SFAs)	1360.36	999.00	1066.20	1080.25	184.10	0.285
Unsaturated fatty acids (UFAs)	1484.58	1174.28	1181.73	1076.77	262.57	0.480
Monounsaturated fatty acids	682.55	512.27	531.01	525.62	109.60	0.415
Polyunsaturated fatty acids (PUFAs)	623.30	514.89	483.84	428.54	134.00	0.553
n6/n3	10.55	9.13	10.22	7.15	1.49	0.178
n-3 Polyunsaturated fatty acids (n-3 PUFAs)	53.89	53.54	43.08	51.99	12.14	0.789
n-6 Polyunsaturated fatty acids (n-6 PUFAs)	569.41	461.35	440.76	376.55	123.75	0.509

CON, basal diet; TP2, basal diet + 2 g/kg tea polyphenols; TP4, basal diet + 4 g/kg tea polyphenols; TP6, basal diet + 6 g/kg tea polyphenols; n6/n3, n-6 polyunsaturated fatty acids/n-3 polyunsaturated fatty acids. SEM: the standard error of the mean. ^a,b^ Values in the same row with different letters are significantly different (*p* < 0.05). The results are presented as the means ± SEMs (*n* = 10).

## Data Availability

The data analyzed in this study are available from the corresponding author upon request.
